# Protein nutrition governs within-host race of honey bee pathogens

**DOI:** 10.1038/s41598-017-15358-w

**Published:** 2017-11-08

**Authors:** Manuel Tritschler, Jutta J. Vollmann, Orlando Yañez, Nor Chejanovsky, Karl Crailsheim, Peter Neumann

**Affiliations:** 1 Institute of Bee Health, Vetsuisse Faculty, University of Bern, Bern, Switzerland; 2 Chemisches und Veterinäruntersuchungsamt Freiburg (CVUA), Bienengesundheit, 79108 Freiburg i. Br. Germany; 3 Institute for Zoology, University of Graz, Graz, Austria; 4 Institute of Plant Protection, The Agricultural Research Organization, The Volcani Center, Rishon LeTsiyon, Israel; 5 Swiss Bee Research Centre, Agroscope, Bern, Switzerland

## Abstract

Multiple infections are common in honey bees, *Apis mellifera*, but the possible role of nutrition in this regard is poorly understood. Microsporidian infections, which are promoted by protein-fed, can negatively correlate with virus infections, but the role of protein nutrition for the microsporidian-virus interface is unknown. Here, we challenged naturally deformed wing virus - B (DWV-B) infected adult honey bee workers fed with or without pollen ( = protein) in hoarding cages, with the microsporidian *Nosema ceranae*. Bee mortality was recorded for 14 days and *N. ceranae* spore loads and DWV-B titers were quantified. Amongst the groups inoculated with *N. ceranae*, more spores were counted in protein-fed bees. However, *N. ceranae* infected bees without protein-diet had reduced longevity compared to all other groups. *N. ceranae* infection had no effect on protein-fed bee’s longevity, whereas bees supplied only with sugar-water showed reduced survival. Our data also support that protein-feeding can have a significant negative impact on virus infections in insects. The negative correlation between *N. ceranae* spore loads and DWV-B titers was stronger expressed in protein-fed hosts. Proteins not only enhance survival of infected hosts, but also significantly shape the microsporidian-virus interface, probably due to increased spore production and enhanced host immunity.

## Introduction

Host nutrition can play a key role for the outcome of pathogen infections in humans and animals^1^, since it is critical for immune-defense and resistance to pathogens^2^. Poor nutrition, in particular protein depletion, is a major factor in high incidence and mortality due to infectious diseases^2,3^.

In insects, the role of nutrition for the outcome of infections is less well understood^4^. The importance of proteins for pathogen resistance has been suggested in caterpillars, *Spodoptera littoralis*, where their resistance to viral infection increased as the protein to carbohydrate ratio in their diet increased^5^. Moreover, infected larvae of the African moth, *Spodoptera exempta*, select a higher protein diet, suggesting that nutrition has a self-medication value^6^.

Feeding protein to honey bee, *Apis mellifera*, workers infected with microsporidian endoparasites, *Nosema apis*, resulted in increased spore development, but also improved the longevity of infected hosts^7^. Similar findings were reported for *Nosema ceranae* ^8^. In bumblebees, *Bombus terrestris*, protein deprivation nutrition can functionally alter not only general resistance, but also alter the pattern of specific host–parasite interactions, probably due to reduced immune responses^9^.

Since hosts infected by more than one pathogen are common, pathogen-pathogen interactions require more attention^10–13^. This is especially true for managed honey bees, *A. mellifera*, which are exposed to a long list of pathogens, which can act as drivers for colony losses especially in areas with established ectoparasitic mite, *Varroa destructor*, populations^14,15^. Since many honey bee pathogens are ubiquitous^16^ multiple viral, fungal and bacterial infections of colonies and even individual bees are most likely and can result in lethal effects to the host^14^.

However, the actual outcome of such multiple infections depends on the nature of interactions between the pathogens in one host. These parasite-parasite interactions in infected individual honey bee hosts can potentially range from competition to cooperation^17–22^.

For example, *V. destructor* is intimately associated with viruses, e.g. deformed wing virus (DWV)^23–25^, especially because it is a very efficient virus vector, generating a disease epidemic within the colony, which dwindles until it dies^26^. *V. destructor* can also activate latent virus infections^27^. On the other hand, there is evidence for antagonistic interactions between honey bee parasites, e.g. between the microsporidians *Nosema ceranae* and *Nosema apis* ^28^, *N. ceranae* and DWV^29,30^. Synergistic effects have been reported between *N. apis* and several viruses, e.g. filamentous virus, bee virus Y and black queen cell virus (BQCV)^31,32^. In contrast, no association was found between Israel acute paralysis virus (IAPV) and *Nosema ceranae* ^33^. This large range of possible interactions between pathogens in one host calls for investigation of possible mechanisms driving this interface.

Since it is known that proteins can impact both virus and microsporidian infections in insects, we regard it as likely that this will affect the outcome of virus-microsporidian interactions in multiple infected hosts. Pollen is the main natural source of protein for honey bees, especially for young bees. It is an essential protein source and may interfere with pathogen-pathogen interactions, infection intensity and longevity of the honey bee host^34,35^.

Here, we investigated the possible role of protein feed via pollen on the interface between DWV-B (formerly *Varroa destructor virus*-1) and *Nosema ceranae* in individual honey bee workers. We hypothesize that protein fed in the form of pollen will not only have significant beneficial effects for the hosts, but will also amplify the virus-microsporidian interface.

## Results

Since the experimental pollen was not irradiated, some bees (N = 19) of the Pollen-only treatment were naturally infected with *N. ceranae*. These contaminated bees were excluded from further data analyses.

The virus strain-specific PCR^36^ showed that only DWV-B was infesting the experimental bees. Neither BQCV, DWV, nor acute bee paralysis virus (ABPV) were found in any of the analyzed individual honey bee workers (N = 120).

All data (*N. ceranae* spore loads, DWV-B infection levels, sugar and pollen consumption) were not normally distributed (Shapiro-Wilk’s test for normality, P < 0.05 in all cases). Therefore, the non-parametric Kruskal-Wallis multiple comparisons One Way ANOVA, Dunn’s Tests were performed.

**Figure 1:**
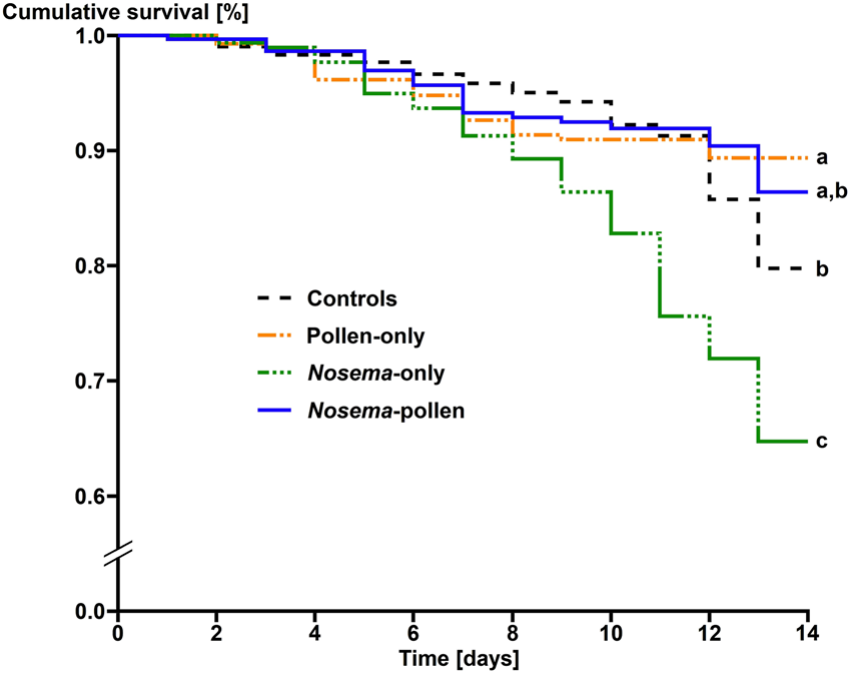
Cumulative survival of honey bee workers exposed to the different treatments over time. Workers contaminated with *N. ceranae* from the pollen-only treatment are not considered. Significant differences (Log-Rank test P < 0.05) among treatments are indicated by different letters (a, b, c).

The survival in the different groups is shown in (Fig. 1). While a significantly reduced longevity was observed for *Nosema-*only infected bees compared to all other groups (Kaplan-Meier, Log-Rank test, P < 0.05), *N. ceranae* infection had no significant effect on the longevity of pollen-fed bees (Kaplan-Meier, Log-Rank test, P > 0.05). Bees that were supplied only with sugar water showed a reduced survival compared to the bees which received both sugar and pollen (Kaplan-Meier, Log-Rank test, P < 0.05, Fig. 1). Hence, workers exposed to both *N. ceranae* and pollen showed a non-additive effect when compared to both treatments individually (χ^2^ = 22.73 (equation-3), theoretical χ^2^ = 7.879, df = 1, P = 0.005). Due to the calculated negative value −33.87 (equation-4), the observed effect on mortality can be considered antagonistic.

**Figure 2:**
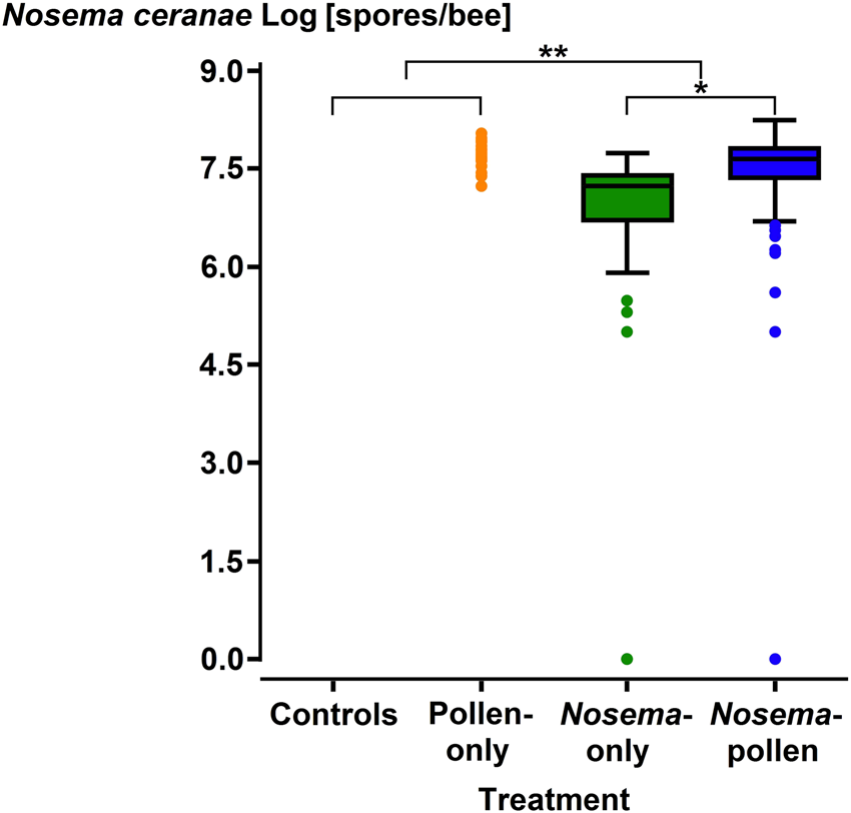
*N. ceranae* spore loads of individual honey bee workers in the four treatment groups (Controls, Pollen-only [orange], *Nosema*-only [green], *Nosema*-pollen [blue]). Medians, ranges, confidence intervals and outliers ( = dots) are shown at a log scale. Significant differences were found between the groups *Nosema*-pollen and *Nosema*-only, as well as between the two *Nosema* groups and the Controls. Please note that N = 19 bees in the Pollen-only treatment were naturally contaminated with *N. ceranae*. When excluding these contaminated bees from the Pollen-only group, significant differences were still found between the groups *Nosema*-pollen and *Nosema*-only (*P = 0.025, **P < 0.0001).

Among the four treatments (Control; Pollen-only; *Nosema*-only; *Nosema*-pollen) significant differences in *N. ceranae* spore loads were only found between *Nosema*-only and *Nosema*-pollen groups (Kruskal-Wallis multiple comparisons One Way ANOVA, Dunn’s Test z > 2.64, P = 0.025, Fig. 2). Non-inoculated bees from the control group showed no *N. ceranae* infections.

**Figure 3:**
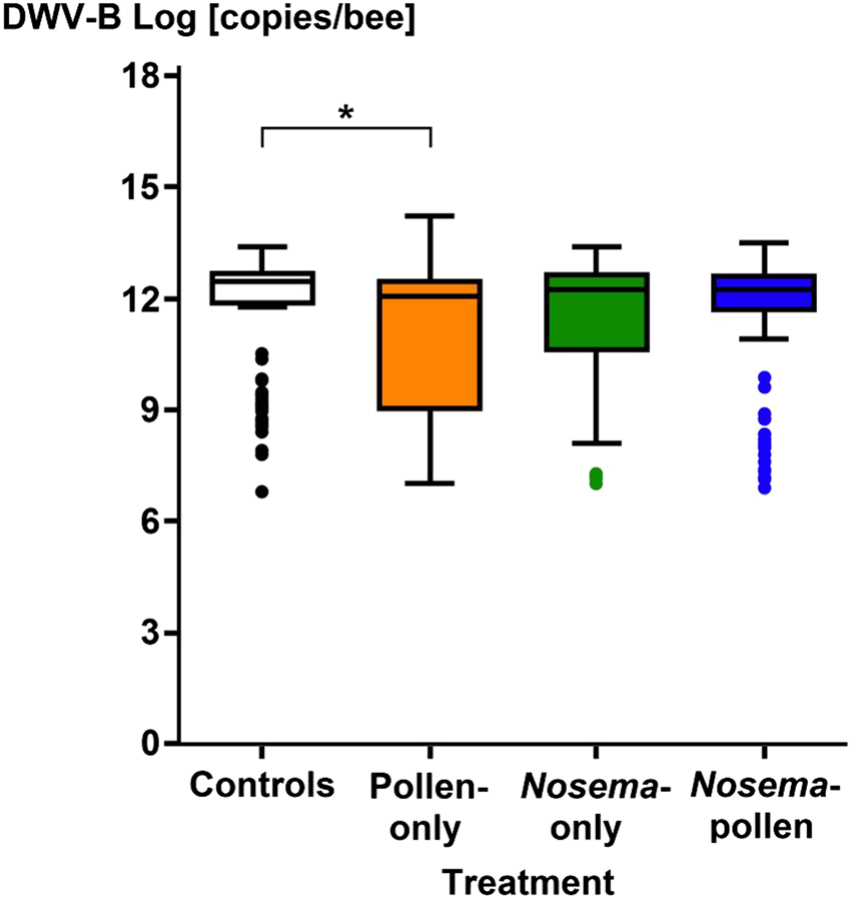
DWV-B infection loads of individual honey bee workers in the four treatment groups (Controls [white], Pollen-only [orange] *Nosema*-only [green], *Nosema*-pollen [blue]). Medians, ranges, confidence intervals and outliers ( = dots) are shown at a log scale. Workers contaminated with *N. ceranae* from the pollen-only treatment are not considered. Significant differences were found between the controls and the Pollen-only group (*P < 0.0001).

The naturally occurring DWV-B infections were significantly different between the four treatment groups (Kruskal-Wallis multiple comparisons One Way ANOVA, Dunn’s Test z > 2.64, P = 0.006, Fig. 3). A significant higher virus load was observed in the Control group compared to the Pollen-only treatment (Kruskal-Wallis multiple comparisons One Way ANOVA, Dunn’s Test * = z > 1.96, P < 0.0001). There were no significant differences between the other groups: (i) for Control and *Nosema*-only (Kruskal-Wallis multiple comparisons One Way ANOVA, Dunn’s Test z > 1.96, P = 0.14), (ii) Controls and *Nosema*-pollen (Kruskal-Wallis multiple comparisons One Way ANOVA, Dunn’s Test z > 1.96, P = 0.1), (iii) Pollen-only and *Nosema*-only (Kruskal-Wallis multiple comparisons One Way ANOVA, Dunn’s Test z > 1.96, P = 0.22), (iv) Pollen-only and *Nosema*-pollen (Kruskal- Wallis multiple comparisons One Way ANOVA, Dunn’s Test z > 1.96, P = 0.06), (v) *Nosema*-only and *Nosema*-pollen (Kruskal-Wallis multiple comparisons One Way ANOVA, Dunn’s Test z > 1.96, P = 0.75).

**Figure 4:**
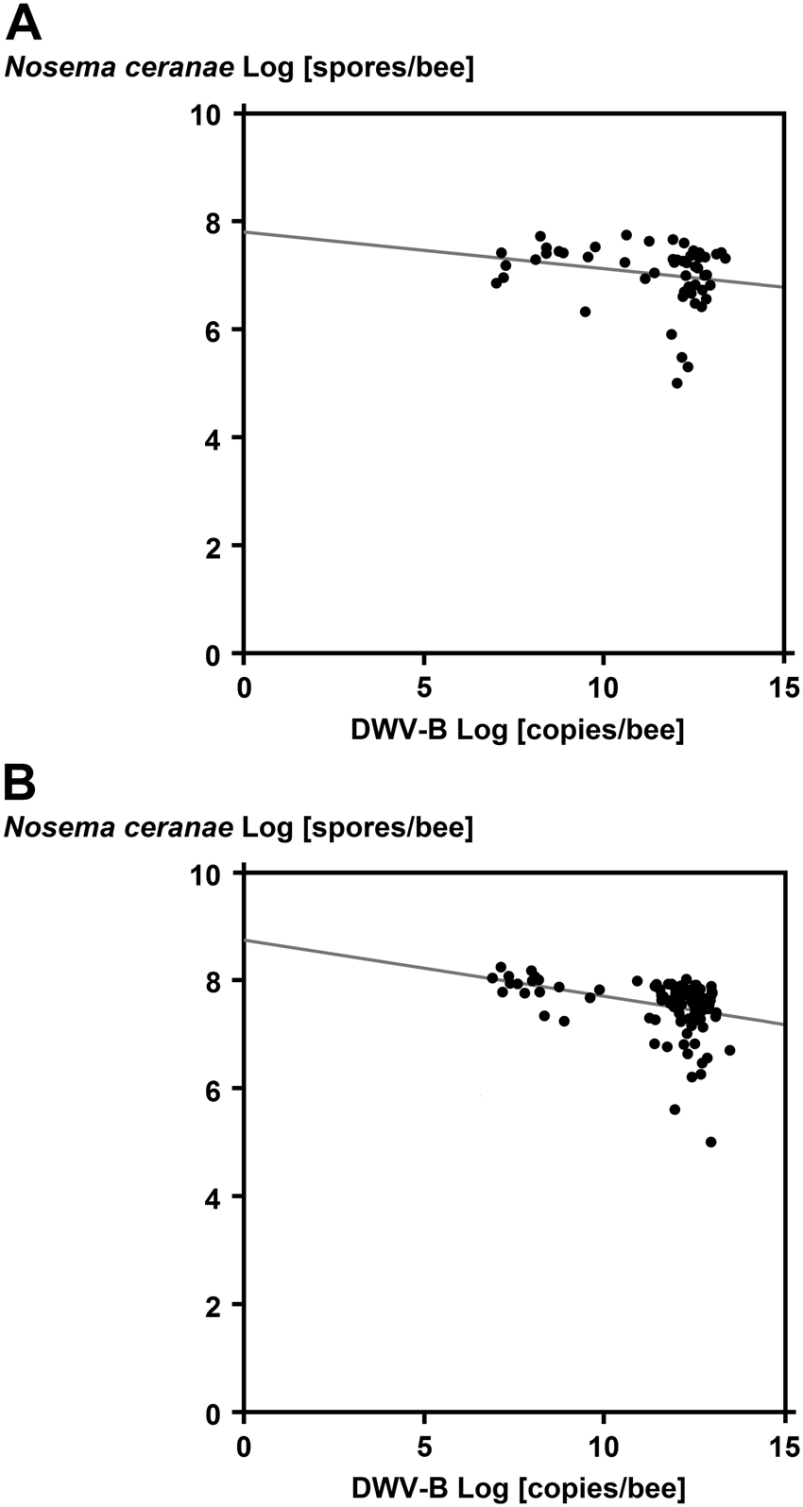
Correlations between *N ceranae* spore loads per bee and DWV-B copy numbers per bee in the treatment groups *Nosema*-only (**A**) and *Nosema*-pollen (**B**) at a log scale. While there was no significant correlation in the *Nosema*-only treatment (Pearson Correlation, Scatter Plot |r| = −0.22, P = 0.12), a highly significant negative correlation was found in the *Nosema*-pollen treatment (Pearson Correlation, Scatter Plot |r| = −0.34, P = 0.0035).

The correlation between *N. ceranae* spore loads and DWV-B infection levels was not significant in the *Nosema*-only treatment (Pearson Correlation: Pearson |r| = −0.22, P = 0.12; Fig. 4). However, a significant negative correlation was found between *N. ceranae* spore loads and DWV-B infection levels in the *Nosema*-pollen treatment (Pearson Correlation: Pearson |r| = −0.34, P = 0.0035). The expected interaction of virus for combined agents was calculated between *N. ceranae* and DWV-B in the combined treatments (*Nosema*-only and *Nosema*-pollen) and can be considered close to antagonistic due to the calculated negative value −10.58 (equation-5) between the two pathogens (χ^2^ = 4.905 (equation-3), theoretical χ^2^ = 3.841, df = 1, P = 0.05). However, the interactive effects of *N. ceranae* in the combined treatments (Pollen-only, *Nosema*-only and *Nosema*-pollen) is close to additive due to the calculated smaller χ^2^-value (χ^2^ = 0.025 (equation-3), theoretical χ^2^ = 3.841, df = 1, P = 0.05).

**Figure 5:**
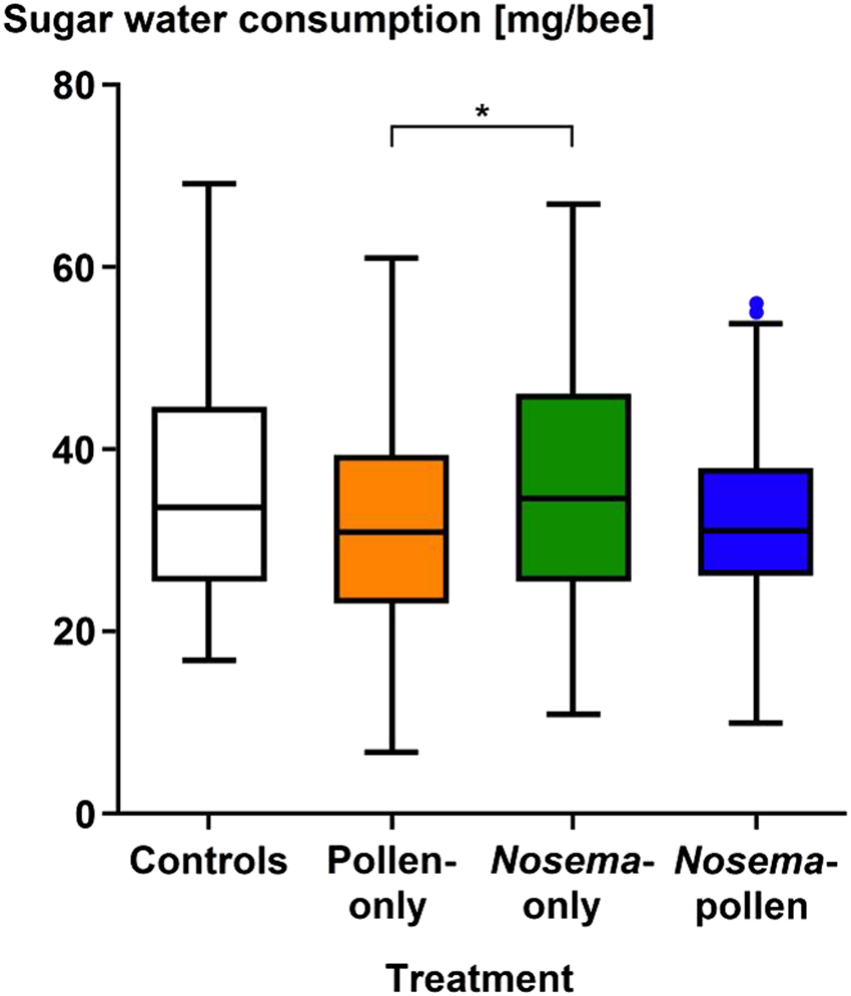
Sugar water consumption in [mg/bee] of individual honey bee workers in the four treatment groups (Controls [white], Pollen-only [orange] *Nosema*-only [green], *Nosema*-pollen [blue]). Workers contaminated with *N. ceranae* from the pollen-only treatment are not considered. Medians, ranges, confidence intervals and outliers ( = dots). Significant differences were found between the Pollen-only and *Nosema*-only group (*P = 0.0089).

The daily sugar water consumption was significantly different between the four treatments (Kruskal-Wallis multiple comparisons One Way ANOVA, Dunn’s Test z > 2.64, P = 0.017, Fig. 5). A significantly higher sugar water consumption was observed for the *Nosema*-only group compared to the Pollen-only group (Kruskal-Wallis multiple comparisons One Way ANOVA, Dunn’s Test * = z > 1.96, P = 0.0089). No significant differences in sugar water consumption were observed between the other groups: (i) Controls and Pollen-only (Kruskal- Wallis multiple comparisons One Way ANOVA, Dunn’s Test z > 1.96, P = 0.09), (ii) Controls and *Nosema*-only (Kruskal-Wallis multiple comparisons One Way ANOVA, Dunn’s Test z > 1.96, P = 0.79), (iii) Controls and *Nosema*-pollen (Kruskal-Wallis multiple comparisons One Way ANOVA, Dunn’s Test z > 1.96, P = 0.1), (iv) Pollen-only and *Nosema*-pollen (Kruskal-Wallis multiple comparisons One Way ANOVA, Dunn’s Test z > 1.96, P = 0.3) and (v) *Nosema*-only and *Nosema*-pollen (Kruskal-Wallis multiple comparisons One Way ANOVA, Dunn’s Test z > 1.96, P = 0.082).

**Figure 6:**
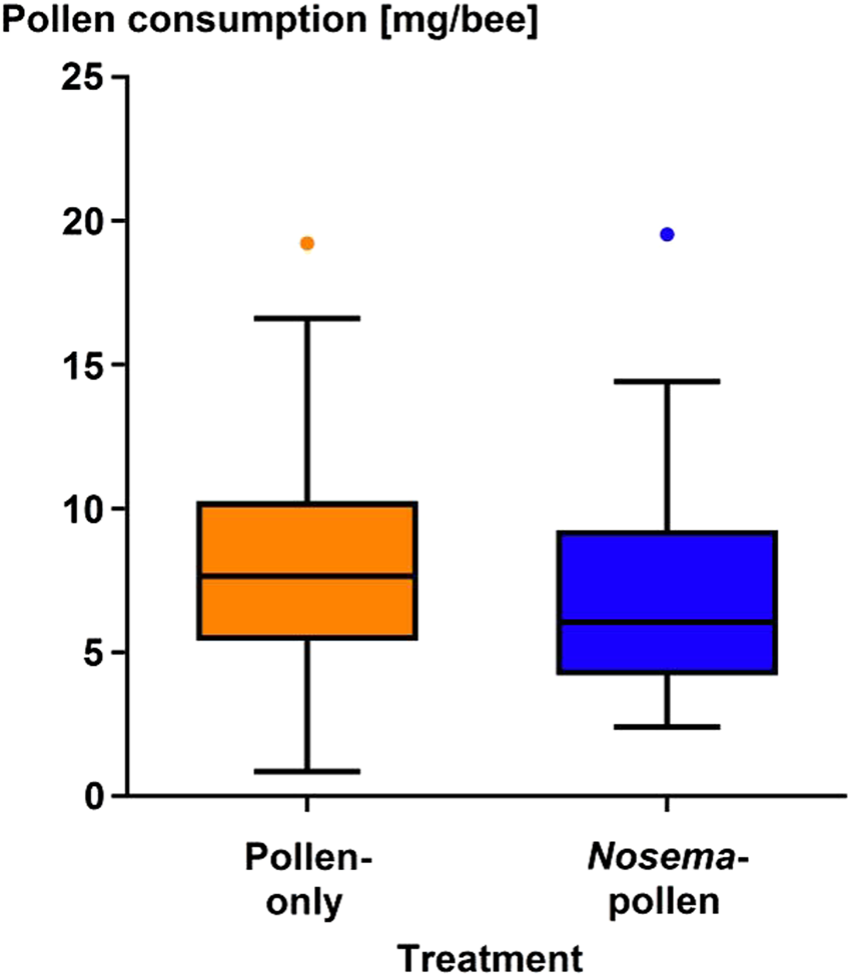
Pollen consumption [mg/bee] of individual honey bee workers in the two pollen treated groups (Pollen-only [orange], *Nosema*-pollen [blue]). Medians, ranges, confidence intervals and outliers ( = dots) are shown. Workers contaminated with *N. ceranae* from the pollen-only treatment are not considered. No significant differences were found between the Pollen-only and the *Nosema*-pollen group (P > 0.05).

The two pollen-fed treatments (Pollen-only, *Nosema*-pollen) showed no significant difference in pollen consumption over the 14 days (Kruskal-Wallis multiple comparisons One Way ANOVA, Dunn’s Test z > 1.96, P > 0.05, Fig. 6).

## Discussion

Our data show for the first time that protein-feeding can have a significant impact on the microsporidian-virus interface in double-infected insect hosts. Taken together with the here confirmed impact on microsporidian^8^ and virus^5,37^ infections and on host survival, our results also provide strong support that protein nutrition can functionally alter not only general resistance in insects, but also alter the pattern of host–parasite interactions^9^.

The experimental pollen was not sterilized similar to other studies^8^. Therefore, some of the Pollen-only treated workers in our experiment became naturally infected with *N. ceranae*. These bees were excluded from any further data analyses. Due to possibly associated probiotic microorganisms^38^, such non-irradiated pollen is probably more beneficial than irradiated one and thus more likely to reveal the full potential of adequate pollen-fed for honey bee resilience. It also appears likely that bees from the *Nosema*-pollen treatment have obtained additional spores. However, this seems not relevant due to the very high number of *N. ceranae* spores used for the treatments.

Our data confirm that *Nosema* spp. infected honey bee workers display higher spore loads when they are pollen-fed (*N. apis*:^6^; *N. ceranae*:^7,39,40^). This is most likely because *Nosema* spp. are highly dependent on host nutritional status for their own development, e.g. host amino acids^41,42^, Adenosine triphosphate (ATP)^43,44^ or other core nutrients^45^. Therefore, it appears obvious that any individual host with a pollen-rich diet becomes ideal for *Nosema* spp. reproduction simply by providing a supreme nutritional environment. However, since protein fed is likely to enhance the immune system^5,9^, the question emerges, why the bees were not able to reduce the *N. ceranae* spore load. This might be explained by the lack of host-parasite co-evolution between *A. mellifera* and the fairly recent invasive species *N. ceranae*. This scenario seems likely because beekeepers limit natural selection, thereby preventing adaptation of honey bees to this and other novel parasites (i.e. *Varroa destructor*)^46^. Alternatively, but not mutually exclusive, *N. ceranae* might have interfered with the host immune response^47^. Nevertheless, despite higher spore loads these *N. ceranae* infected individuals showed an improved survival confirming earlier findings^8^. Adequate pollen availability might compensate for the energy and nutrients lost in honey bees with high *N. ceranae* infection intensity, thereby enabling improved survival^8^. These results are also in line with another study^48^ that nutrition influences survival in colony level *N. ceranae* infections. In a simultaneous choice test between sunflower honeys and honeydew *N. ceranae* infected bees significantly preferred sunflower honey over honey dew^49^ Such bees consuming sunflower honey showed significantly lower *N. ceranae* spore loads compared to the honeydew group, probably because of the higher antibiotic activity of sun flower honey^49^. These findings are well in line with therapeutic self-medication reported from primates^50^ and butterflies^51^.

Our data also provide support to the key role of protein nutrition for the outcome of virus infections in insect hosts^5,37^. Indeed, bees only receiving sugar water showed a significantly higher mortality and higher DWV-B infection levels compared to pollen-fed ones. This confirms that DWV infected bees fed with pollen show lower viral loads than bees only fed with sugar water^52^. Since just a subsample of 30 bees per treatment was tested for other viruses (BQCV, DWV, ABPV), it can obviously not be excluded that those viruses might also have contributed to a higher bee mortality. However, none of the other viruses were found in any of the analyzed honey bees suggesting a high probability for their absence.

Most interestingly, the results show for the first time that protein-feeding can significantly impact the microsporidian-virus interface in double-infected insect hosts. What are possible reasons for this shifted pathogen interface? In line with other studies^30^, pre-infection of DWV did not interfere with *N. ceranae* replication, but *N. ceranae* did interfere with DWV replication. Since *N. ceranae* replication in midgut cells disrupts protein metabolism and causes energetic stress^8,53,54^, this microsporidian is likely to compete with orally acquired viruses for cell resources or for DWV-B’s accessibility to midgut cells^30^. This could explain why an increase in cell resources due to better protein fed led to higher *N. ceranae* spore counts, but also resulted in lower virus loads. Moreover, *N. ceranae* was shown to induce a significant increase in phenol oxidase in bees fed on sugar and workers’ longevity in cages was positively linked to phenol oxidase activity^55^. This suggests a second possible explanation for the stronger negative correlation between *N. ceranae* and DWV-B in protein-fed hosts because both phenol oxidase and upregulation of antimicrobial peptides are linked with effective antiviral responses in *A. mellifera* ^56^. Since pollen promotes development of the main honey bee immune organ, the fat body^57^, its maturation may enable a better performance of the immune system including activation of phenol oxidase. Indeed, pollen diet promotes fat body development and enhances survival of *N. ceranae* parasitized workers that have expressed higher levels of vitellogenin and immunoprotein^55,58–61^. Moreover, it is known that pollen supplies are in general immensely important for overwintering honey bee colonies to effectively oppose pathogen stress^40^. Interestingly, Spaetzle, an activator of the Toll pathway was upregulated by pollen feeding in healthy bees as well as a gene coding for the antimicrobial peptide Defensin and the Peptidoglycan recognition protein PGRP-LC^62^. In conclusion, the conjunction of increased *N. ceranae* spore production and enhanced host immunity acquired by proficient protein nutrition most likely explains the observed significant increase in the negative correlation between *N. ceranae* and DWV-B loads. Alternatively, but not mutually exclusive, the negative correlations between *N. ceranae* and DWV infections as found in this study and in others^29,30,63^ can also be explained by individual workers being more susceptible to microsporidians, but less susceptible to virus infections. For example, some bees may be more susceptible to *Nosema* spp. infections, but could be better able to resist viruses resulting in an overall significant correlation. Indeed, a genetic basis for disease susceptibility is long known in honey bees^64^, incl. *Nosema* spp. infections^65^, and colonies consist of a large number of subfamilies, so-called patrilines, due to the high degree of polyandry by queens^66^. This provides an alternative explanation for the observed negative correlation between the two pathogens in individual hosts without assuming any antagonistic interactions between them.

No differences in the amount of consumed sugar water were found between control and the Pollen-only group compared to the two *N. ceranae* -treated groups similar to an earlier study^8^. The higher amounts of sugar-water consumption by *N. ceranae*-infected bees observed in other studies^67–69^ may be linked to the lack of protein fed in the respective experimental designs. Our study also revealed a significantly higher sugar water consumption in the *Nosema*-only group compared to Pollen-only workers, suggesting that lack of protein combined with microsporidian infections may result in higher hunger levels^67^. In line with an earlier study^8^, we found no significant differences in the amount of pollen consumed between the Pollen-only and the *Nosema*-pollen groups. Even though *N. ceranae* infections certainly induces costs for the hosts, those do apparently not result in significant differences in protein consumption between infected and non-infected bees at least over our experimental period. In conclusion, irrespective of the actual mechanisms underlying the observed stronger negative correlation between microsporidian and virus infection levels in protein-fed hosts, our data strongly suggest that proteins can govern the pathogen-pathogen interface in double-infected insect hosts. Our results further provide support that protein nutrition is an overall key factor for the outcome of infections in insects^5–9^.

## Material and Methods

### Study design

The experiment was conducted in September 2014 at the Institute of Zoology, Karl-Franzens University, Graz, Austria using honey bee workers from four randomly chosen queenright local colonies (predominantly *A. m. carnica*). All colonies were routinely treated against *V. destructor* in late summer using formic acid and oxalic acid in the previous winter^70–72^.

To test whether pollen nutrition has an effect on *N. ceranae* interactions with naturally occurring virus infections, a fully-crossed hoarding cage experiment was performed with four replicates each: 1. Workers fed with sugar, but not with pollen (=Controls); 2. Workers fed with both sugar and pollen (=Pollen-only); 3. Workers fed with *N. ceranae* spores and sugar, but without pollen (=*Nosema*-only); 4. Workers fed with *N. ceranae* spores, sugar and pollen (=*Nosema*-pollen).

### Spore solutions

The *N. ceranae* spore solutions were prepared following routine protocols^73^. In brief, 12 foragers were collected from the hive entrances of four local infested colonies and dissected. Then, three midguts of *N. ceranae* infested workers were pooled together in a vial with 0.5 ml water. After homogenization, each vial solution was checked under the light microscope (x400 objective) for the presence of *N. ceranae* spores^74^. After all four vials were checked for positive spore loads; the spore solutions were mixed and centrifuged at 5 000 rpm for 5 min. The supernatant containing tissue debris was discarded and the spore pellet was re-suspended in 0.5 ml water by vortexing for 5 sec until spores were uniformly distributed in the solution. This washing step was repeated twice until the *N. ceranae* spore solution had a concentration of at least 85% purity^73^. The re-suspended solution was 500 µl water in a 1.5 ml Eppendorf^®^ tube prior to spore load quantification using a haemocytometer and light microscopy (Thermo Fisher Scientific, Waltham, Massachusetts, USA)^75,76^ focusing on five large squares (each containing 16 small squares) in which the *N. ceranae* spores were counted. The final concentrations of the spore solutions were quantified using the following calculation^77^:

1 $${S}_{N}={S}_{H}\ast 50.000$$

where S_N_ is the number of spores per honey bee in 500 µl and S_H_ is the number of spores in 5 large haemocytometer squares (80 small squares). The taxonomic status of the spores was confirmed using *N. ceranae* species-specific PCR^73^ for 30 individual honey bees of each treatment.

### Experimental set up

Four frames with sealed worker brood were taken from each of the four experimental colonies and placed in an incubator at (34.5 °C) until adult emergence. To ensure that the bees were not older than 24 h, all bees on the brood frames were removed the evening before the experiment started. Each treatment group consisted of six standard hoarding cages with 50 workers randomly assigned to each cage^78^. Bees were fed with 50% sugar water (w/v) *ad libitum* until the 3^rd^ day^28^, at which pollen feeding and *N. ceranae* infection started. Prior to the treatment, all bees were starved for 2 h^29,53,75^ before *N. ceranae* infection was done by bulk feeding^73^ over 24 h. Workers of the two *N. ceranae* treatments were challenged with ∼100’000 spores per bee^75,78–80^. All workers were fed until the end of the experiment (14 days) *ad libitum* with 50% sugar water (w/v). In addition, pollen-treated bees were provided with pollen dough containing corbicula pollen and sucrose candy^78^. Prior to the experiments, this pollen was not gamma ray irradiated. The experiment lasted 14 d, in which the honey bees were kept in an incubator at brood nest temperature (34.5 °C)^81^ with 75% RH^82^, for the first 6 days, before the temperature was decreased to 30 °C^78^ for the remaining experimental period of 8 days.

To test for potential differences in nutritional demand, pollen dough and sugar water consumption was measured in all cages on a daily basis^67^. The syringes prepared as feeders were refilled every other day in order to avoid the formation of mold^78^. Dead bees were removed daily^76,83^. Possible cage effects were expected as random effects, whereas all replicates had the same conditions in the incubator, temperature and random mixture of bees.

### *N. ceranae* infection levels

Fourteen days post treatment (*N. ceranae* life cycle = 14 days^73^ the experiment was terminated and the surviving individuals (N = 401) were separately freeze-killed and stored at −80 °C until further analyses. To test for *N. ceranae* infection levels, the workers were crushed in 2 ml microcentrifuge tubes containing a 5 mm metal beads and 200 µl TN-buffer (1 M Tris; 1 M NaCl) for 30 sec at 25 shakes per sec. Spore counts and calculations were performed as described above.

### Virus infection levels

All 401 bees were individually analyzed for DWV-B. Prior to RNA extraction, individual bees were crushed in 2 ml microcentrifuge tubes containing 5 mm metal beads and 200 µl TN-buffer (10 mM Tris, 10 mM NaCl; pH 7.6). The samples were homogenized with a tissue-lyser for 30 sec at 25 1/s frequency using a Qiagen Retsch® MM 300 mixer mill^84^. Then, the homogenates were centrifuged at 2500 rpm and 50 µl of the supernatant was destined for total RNA extraction using NucleoSpin RNA extraction kit (Macherey-Nagel) following the manufactures guidelines. Reverse transcription was performed using 2 µg of extracted RNA incubated with random hexamer primers for 5 min at 70 °C. Then mixed with 5 µl of 5x buffer, 1.25 µl dNTP (10 mM) and 1 µl M-MLV before incubating the 25 µl volume reaction for 60 min at 37 °C. For virus quantification, 10-fold diluted cDNA was mixed with Kapa SYBR® FAST qPCR Master Mix kit. Briefly, 6 µl 2x reaction buffer, 0.24 µl forward and reverse primers for DWV-B and β-actin (Table 1)^85^ merged with 2.52 µl water and 3 µl template in a total of 12 µl final reaction volume^86^. The real-time qPCR cycling profile consisted of 3 min incubation at 95 °C and 40 cycles of 3 sec at 95 °C for denaturation, 30 sec at 57 °C for annealing and data collection. The melting-curve analysis was performed with the following conditions: 15 s at 95 °C, 55 °C and 95 °C, respectively. Purified DWV-B PCR products of know concentration (10^−3^–10^−6^ ng) were used as standard curves on each individual plate, along with non-template controls (R^2^: 0.992; Slope: −3.198; Intercept: 30.240; PCR efficiency: 2.054). Quantification of the β-actin gene was performed in parallel for each sample as reference gene for DWV-B normalization^85^. A Cq cut-off value (according to the value of the negative control) was used to define the disease status (positive or negative). The ECO Software real-time PCR system (Illumina, San Diego, CA, USA) was used to evaluate the performance of the qPCR reactions and to analyze the qPCR quantification. These Cq-values were used to calculate the virus infection levels in Log [copies/bees] which were then used for the statistical analyses.

**Table 1:** Primers used for the quantification of honey bee viruses by qPCR assays. The targets, primer names, sequences, the product size and references are shown.

Target	Primer	Sequence (5′-3′)	Size [bp]	Reference
β-actin (A.m.)	A.m. Actin q92F	CGT TGT CCC GAG GCT CTT T	66	85
	A.m. Actin q157	TGT CTC ATG AAT ACC GCA AGC T		
Acute bee paralysis virus	ABPV-F6548	TCATACCTGCCGATCAAG	197	94
	KIABPV-B6707	CTGAATAATACTGTGCGTATC		
Black queen cell virus	BQCV-qF7893	AGTGGCGGAGATGTATGC	294	94
	BQCV-qB8150	GGAGGTGAAGTGGCTATATC		
Deformed wing virus	DWV-F8668	TTCATTAAAGCCACCTGGAACATC	136	94
	DWV-B8757	TTTCCTCATTAACTGTGTCGTTGA		
Deformed wing virus-B	DWV-B-F2	TAT CTT CAT TAA AAC CGC CAG GCT	140	36
	DWV-B-R2	CTT CCT CAT TAA CTG AGT TGT TGT C		

A further subsample of 30 bees per treatment (N = 5 each cage) was screened to determine if other honey bee viruses were also present in the colonies during the experiment: BQCV, which is known to be associated with *N apis* infection^31,32^ and occurs in 30% of Austrian honey bees^87^. Three more viruses associated with honey bees in Austria were screened: DWV which is present in 91% of Austrian honey bees including its variant DWV-B and ABPV reported to be present in 68% of Austrian bees^87^.

### Statistical analyses

Data were tested for normality using Shapiro-Wilk’s test (P > 0.05). If, however, normality was rejected (Shapiro-Wilk’s test, P < 0.05), groups were compared by performing non-parametric Kruskal-Wallis multiple comparison One Way ANOVAs (Dunn’s test) and Pearson correlation.

Longevity analyses for the four individual treatment groups were conducted by using Kaplan-Meier Survival Curves and a Log-Rank assessment.

Interactions between treatments on worker mortality were determined by using χ^2^ tests^88,89^. The expected interaction mortality value, M_E_ for combined treatment was calculated using the following formula:

2 $${M}_{E}={M}_{PT}+{M}_{N}(1-\frac{{M}_{PT}}{100})$$

where M_PT_ and M_N_ are the observed percent mortalities caused by pollen treatment and *N. ceranae* infection.

The resulting values from each equation were then compared to the χ^2^ table value with 1 df, using the formula:

3 $${\chi }^{2}=\frac{{({M}_{O}-{M}_{E})}^{2}}{{M}_{E}}$$

where M_*O*_ is the observed mortality for the combined *N. ceranae* with pollen treatment.

A non-additive effect between the two agents was expected when the χ^2^ value exceeded the given table value. If, however the difference between

4 $${M}_{O}-{M}_{E}$$

or

5 $${V}_{O}-{V}_{E}$$

had a positive or a negative value, an interaction was then regarded as being synergistic or antagonistic, respectively^88^.

Synergistic, additive, or antagonistic interactions between agents in the combination treatments for DWV-B loads and *N. ceranae* spores were determined using a χ^2^ test^90–93^.

Comparisons of *N. ceranae* spore loads, DWV-B infection levels and sugar/pollen consumption rates in the different treatment groups were performed using Kruskal-Wallis One Way ANOVAs (and multiple comparisons, Dunn’s Test).

Pearson correlations between *N. ceranae* spore loads and DWV-B infections levels (Log [copies/bee]) were performed for both *Nosema*-only and *Nosema*-pollen groups.

All statistical analyses were performed using the program NCSS (NCSS 9 Statistical Analysis and Graphics).
